# Dexmedetomidine-containing patient-controlled intravenous analgesia after elective caesarean delivery: a retrospective cohort study

**DOI:** 10.3389/fmed.2026.1991923

**Published:** 2026-10-14

**Authors:** YinJun Lu, YuHui Huang, ChaoRen Hu, Li Ying, ShuCan Quan, Lei Li, Xiongying Li, YingLi Cheng

**Affiliations:** 1 Department of Anesthesiology, Yongkang Women and Children's Health Hospital, Yongkang, Zhejiang, China; 2 Department of Laboratory Medicine, Yongkang Women and Children's Health Hospital, Yongkang, Zhejiang, China; 3 Department of Obstetrics, Yongkang Women and Children's Health Hospital, Yongkang, Zhejiang, China

**Keywords:** caesarean delivery, dexmedetomidine, multimodal analgesia, patient-controlled intravenous analgesia, postoperative recovery

## Abstract

**Background:**

Dexmedetomidine is used as an adjunct in multimodal postoperative analgesia, but evidence from routine clinical care after caesarean delivery remains limited. We evaluated whether dexmedetomidine-containing patient-controlled intravenous analgesia (PCIA) was associated with postoperative analgesic and early recovery outcomes.

**Methods:**

This single-centre retrospective cohort study included women aged 18–45 years undergoing elective term caesarean delivery between January and September 2025. Women were classified according to the initial postoperative PCIA regimen as receiving dexmedetomidine-containing or conventional PCIA. The primary outcome was movement pain on a 0–10 numerical rating scale at 24 h. Secondary outcomes included pain through 48 h, sufentanil delivery, patient-controlled analgesia demands, rescue analgesia, treatment-related events, and early recovery milestones. Propensity-score overlap weighting was used for the primary analysis. Propensity-score matching, conventional multivariable adjustment, complete-case and restricted-cohort analyses, and an expanded propensity-score model incorporating attending anaesthesiologist and calendar month were used to assess robustness.

**Results:**

Among 304 eligible women, 132 received dexmedetomidine-containing PCIA and 172 received conventional PCIA. Mean movement pain at 24 h was 3.1 (SD 1.2) versus 3.7 (SD 1.3), corresponding to an overlap-weighted mean difference of −0.51 points (95% CI −0.80 to −0.22; *P* < 0.001). The primary estimate was similar across sensitivity analyses, including the expanded propensity-score model incorporating attending anaesthesiologist and calendar month. Among 290 women with complete 48-h pump records, median cumulative sufentanil delivery was 84 μg (IQR 78–91) versus 90 μg (82–98; median difference −5.2 μg, 95% CI −7.8 to −2.6; *P* < 0.001). Rescue analgesia was required in 24 of 132 women (18.2%) versus 46 of 172 (26.7%; risk ratio 0.72, 95% CI 0.52–0.99; *P* = 0.044). First mobilisation occurred at 16.9 versus 19.3 h (time ratio 0.89, 95% CI 0.84–0.95; *P* < 0.001), and first passage of flatus at 25.8 versus 28.9 h (time ratio 0.92, 95% CI 0.86–0.98; *P* = 0.012). Estimates for uncommon adverse events were imprecise.

**Conclusions:**

In this retrospective cohort, dexmedetomidine-containing PCIA was associated with modestly lower early movement pain and lower analgesic requirements, with shorter observed times to selected early recovery milestones. Estimates for uncommon adverse events did not establish safety equivalence. These observational findings should not be interpreted as establishing a causal treatment effect.

## 1 Introduction

Pain after caesarean delivery is common, clinically relevant, and often most apparent during movement rather than at rest (1). Turning in bed, sitting upright, walking, breastfeeding, and caring for the newborn may all provoke pain during the first postoperative day, when early mobilisation and maternal recovery are expected to begin. Although neuraxial anaesthesia, intrathecal morphine, non-steroidal anti-inflammatory drugs, paracetamol, and regional blocks have improved postoperative analgesia, these measures are not applied uniformly in routine practice, and residual movement pain remains frequent (2, 3). In this setting, pain assessment at 24 h is particularly informative because it captures the period in which analgesic efficacy, opioid exposure, and functional recovery intersect. A single pain score, however, provides only a limited view of postoperative burden. Clinically meaningful evaluation should also consider pain trajectories over 12, 24, and 48 h, actual opioid delivery, patient-controlled analgesia use, rescue treatment, and the timing of early recovery milestones.

Patient-controlled intravenous analgesia remains an important component of postoperative care in many obstetric units, particularly when neuraxial opioid use is limited, contraindicated, or judged insufficient (4). Opioid-based regimens can provide effective analgesia, but their use is constrained by nausea, vomiting, pruritus, sedation, respiratory depression, and delayed gastrointestinal recovery (5). These concerns are especially relevant after caesarean delivery because even modest impairment in alertness or mobility may affect breastfeeding, ambulation, and newborn care. Even when the base PCIA prescription and pump programme are relatively standardised, actual postoperative analgesic exposure may differ because of patient-controlled bolus use, temporary interruption or early discontinuation of the pump, neuraxial opioid use, transversus abdominis plane block, and concomitant non-opioid analgesia (6). Consequently, the prepared opioid dose does not necessarily reflect actual exposure, and treatment comparisons based only on the prescribed regimen may obscure clinically relevant differences.

Dexmedetomidine, a selective *α*2-adrenergic agonist, has been incorporated into multimodal analgesic regimens because of its potential to enhance analgesia and reduce opioid requirements without direct respiratory suppression (7, 8). Previous studies of dexmedetomidine-containing analgesia after caesarean delivery have reported reductions in pain scores or opioid use, but the available evidence has several limitations (9, 10). Many studies have been relatively small, conducted under tightly controlled protocols, or focused on a single pain time point. Dexmedetomidine dose, background opioid regimen, neuraxial morphine use, and co-analgesic treatment have varied substantially across studies. Safety estimates for bradycardia, hypotension, excessive sedation, and early discontinuation have therefore remained imprecise (11). In addition, few studies have examined whether lower pain and opioid exposure are accompanied by earlier mobilisation or gastrointestinal recovery in routine clinical care.

We therefore conducted a single-centre retrospective cohort study among women aged 18–45 years undergoing elective term caesarean delivery at 37 + 0 to 41 + 6 weeks of gestation. We compared the initial postoperative dexmedetomidine-containing and conventional PCIA regimens. The prespecified primary outcome was movement pain on a 0–10 numerical rating scale at 24 h. Secondary analyses examined resting and movement pain through 48 h, time-weighted movement-pain burden, actual sufentanil delivery, effective patient-controlled demands, rescue analgesia, treatment-related events, and early recovery milestones. Propensity-score overlap weighting was used to address measured baseline differences between groups, with sensitivity analyses and exploratory subgroup analyses used to assess the robustness and heterogeneity of the observed association.

## 2 Methods

### 2.1 Study design, setting, data sources, and analytic framework

This single-centre retrospective cohort study included women undergoing elective term caesarean delivery at Yongkang Women and Children's Health Hospital between 1 January and 30 September 2025. Consecutive potentially eligible cases were identified from the hospital electronic medical record and cross-referenced with the anaesthesia information system, obstetric operating records, postoperative nursing records, electronic patient-controlled analgesia pump records, and laboratory information system. Records were linked within the institutional systems using unique hospital identifiers; the analytic dataset was de-identified before statistical analysis and handled in accordance with institutional data-protection requirements. Prespecified variables were extracted using a standardised data form, and clinically implausible values or inconsistencies between data sources were checked against the original records. The cohort assembly and analytic populations are shown in Figure 1.

**Figure 1:**
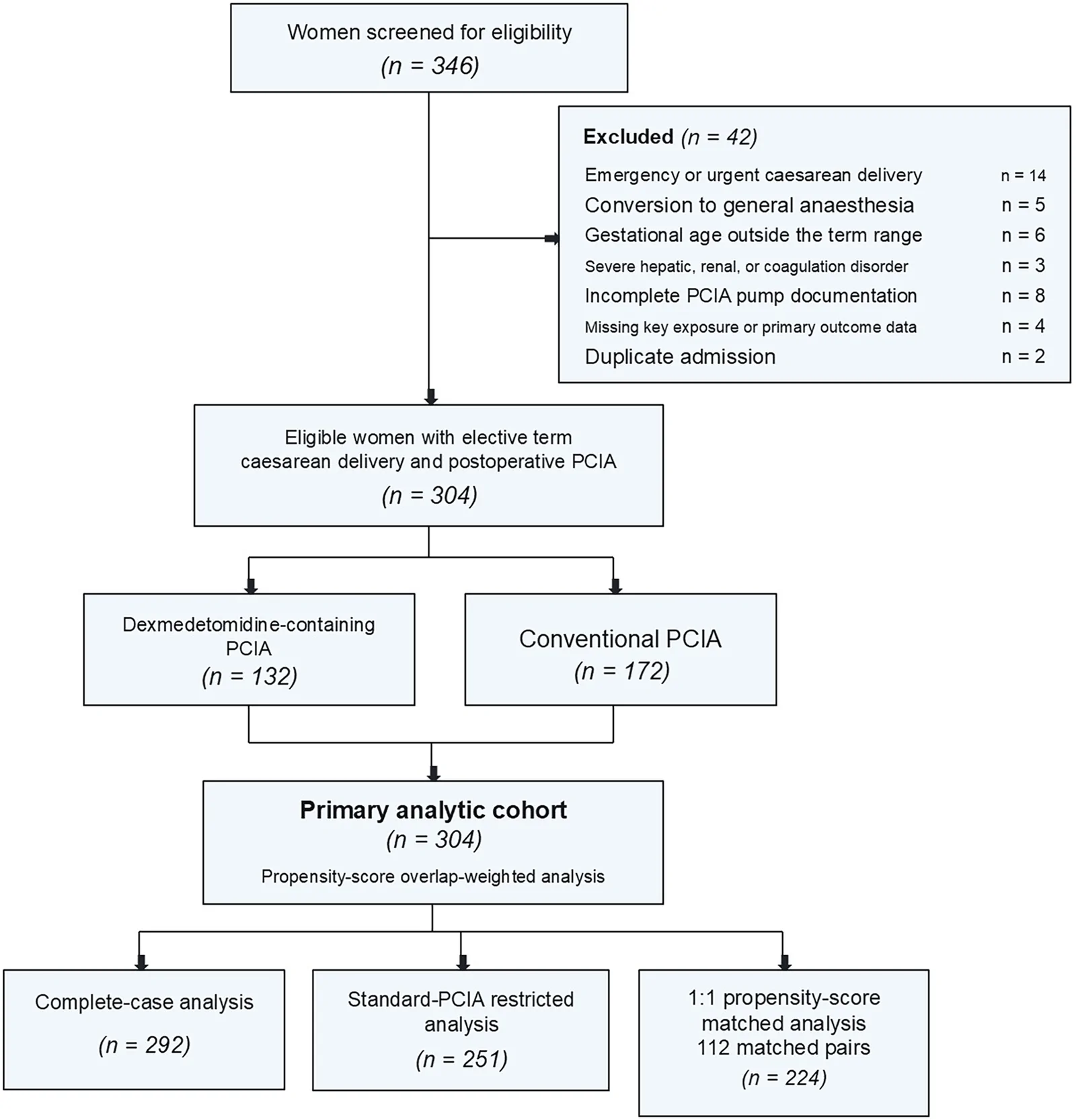
Study flow and construction of the primary analytic cohort. Among 346 women screened, 42 were excluded and 304 women comprised the primary cohort, including 132 who received dexmedetomidine-containing PCIA and 172 who received conventional PCIA. The primary analysis used propensity-score overlap weighting. The complete-case analysis included 292 women, the standard-PCIA restricted analysis included 251 women, and 112 matched pairs were included in the 1:1 propensity-score-matched sensitivity analysis. PCIA, patient-controlled intravenous analgesia.

Eligibility was assessed using information available before initiation of postoperative analgesia. Time zero was defined as completion of surgery and commencement of patient-controlled intravenous analgesia, so that exposure classification and follow-up began at the same postoperative time point in both groups. Women were classified according to the initial postoperative PCIA regimen and remained in that exposure group irrespective of subsequent pump adjustment, temporary interruption, or early discontinuation. Follow-up covered pain and analgesic outcomes during the first 48 postoperative hours and early recovery until discharge; exploratory perioperative coagulation measurements are reported only in the Supplement. The study was approved by the Ethics Committee of Yongkang Women and Children's Health Hospital (Approval No. YFB2024LS035). The requirement for informed consent was waived by the Ethics Committee because of the retrospective nature of the study. All procedures were conducted in accordance with the Declaration of Helsinki.

### 2.2 Participants, eligibility criteria, and cohort assembly

Consecutive women aged 18–45 years who underwent elective caesarean delivery at term, defined as 37 + 0 to 41 + 6 weeks of gestation, were screened. Eligibility required initiation of postoperative patient-controlled intravenous analgesia after surgery and sufficient perioperative documentation to determine the analgesic regimen and the prespecified primary outcome. Gestational age was taken from the final obstetric assessment recorded before delivery, based on first-trimester ultrasonography or the best documented obstetric estimate. When more than one caesarean delivery occurred during the study period, only the first eligible procedure was retained. Women meeting any prespecified exclusion criterion were removed before exposure classification; the complete exclusion categories and corresponding numbers are presented in Figure 1.

Among 346 women screened, 42 were excluded and 304 comprised the primary cohort. Exposure classification was based on the PCIA prescription initiated at time zero, yielding 132 women in the dexmedetomidine-containing group and 172 in the conventional group. The primary analysis retained all eligible women with available 24-h movement pain scores. Secondary analyses used outcome-specific denominators according to data availability. The paired coagulation cohort was formed separately for each laboratory marker and required both a preoperative and postoperative measurement; therefore, sample size varied across platelet count, prothrombin time, activated partial thromboplastin time, fibrinogen, and D-dimer analyses. The propensity score-matched sensitivity cohort included 112 matched pairs. No formal *a priori* sample-size calculation was performed because all eligible women treated during the prespecified period were included. The nine-month study interval was defined by the period during which both PCIA regimens were concurrently available under a stable departmental analgesic pathway and complete electronic PCIA pump records could be systematically retrieved.

### 2.3 Perioperative management and analgesic exposure

Anaesthetic and postoperative analgesic management followed routine departmental practice, and the choice between conventional and dexmedetomidine-containing PCIA was made by the attending anaesthesiologist as part of routine clinical care rather than by random allocation. Both regimens were available throughout the study period, there was no calendar-based transition from one regimen to the other, and no mandatory departmental criterion required use of either regimen. Individual patient-level reasons for regimen selection were not systematically documented and could not be reconstructed retrospectively. Accordingly, regimen assignment was not assumed to be random or quasi-random. Caesarean delivery was performed under neuraxial anaesthesia using either epidural anaesthesia or combined spinal–epidural anaesthesia; standalone spinal anaesthesia was not used in the study cohort. Intrathecal morphine administered during combined spinal–epidural anaesthesia and transversus abdominis plane block, when used, were recorded as baseline perioperative covariates; epidural morphine was not used in this cohort. After surgery, PCIA was initiated according to the regimen selected by the attending anaesthesiologist within the routine departmental analgesic pathway. Concomitant non-opioid analgesics, primarily non-steroidal anti-inflammatory drugs and paracetamol, were administered according to routine clinical indications and contraindications.

The standard conventional PCIA formulation contained sufentanil 100 μg, dezocine 10 mg, and ondansetron 16 mg in a total volume of 100 mL. The standard dexmedetomidine-containing formulation used the same base regimen with dexmedetomidine 100 μg added to the 100-mL solution. The standard pump programme consisted of a background infusion of 1.5 mL/h, a patient-controlled bolus of 2.0 mL, and a 20-min lockout interval. The complete standard base formulation and pump programme were used in 251 women; deviations from the standard formulation or programme were addressed in a restricted-cohort sensitivity analysis. Exposure classification was based on the PCIA prescription initiated at time zero. Subsequent pump adjustment, temporary interruption, or early discontinuation did not alter the original exposure classification. Actual sufentanil delivery and effective patient-controlled demands were derived from electronic pump records rather than from the prepared dose alone. Intraoperative dexmedetomidine administration was recorded separately from postoperative PCIA exposure and was addressed in a sensitivity analysis.

### 2.4 Data collection, covariates, and measurement procedures

Demographic and obstetric variables were obtained from the electronic medical record, while anaesthetic, operative, and analgesic data were extracted from the anaesthesia information system, operating-room records, medication charts, and electronic PCIA pump logs. Prespecified baseline covariates included maternal age, body mass index, gestational age, parity, previous caesarean delivery, gestational diabetes, hypertensive disorders of pregnancy, American Society of Anesthesiologists physical status, anaesthetic technique (epidural vs combined spinal–epidural anaesthesia), intrathecal morphine, transversus abdominis plane block, pre-existing chronic pain or regular analgesic use for more than 3 months, duration of surgery, estimated blood loss, and preoperative haemoglobin and platelet count. Variables were defined using information recorded before initiation of postoperative PCIA. When duplicate entries were present, the value closest to time zero was retained after comparison with the source record.

Pain was assessed using an 11-point numerical rating scale ranging from 0, no pain, to 10, worst imaginable pain (12). Resting and movement pain were recorded separately at approximately 12, 24, and 48 h after surgery as part of routine postoperative assessments performed by 18 obstetric ward nurses using the same clinical documentation framework. The assessments were conducted for routine clinical care rather than for the subsequent retrospective study, although the nurses were not blinded to the PCIA regimen documented in the medical record. No independent duplicate pain ratings were obtained; therefore, inter-rater reliability could not be estimated. When more than one value was available within the same assessment window, the measurement closest to the nominal time was used. Actual sufentanil delivery and effective patient-controlled demands were obtained directly from pump records. Recovery milestones were abstracted from time-stamped nursing documentation. Preoperative coagulation tests were taken from the most recent sample obtained before surgery, and postoperative measurements from the first clinically available sample after surgery. Platelet count was measured using an automated haematology analyser, while prothrombin time, activated partial thromboplastin time, fibrinogen, and D-dimer were measured using standard automated coagulation assays. For the D-dimer sensitivity analysis, postoperative sampling was restricted to 24 ± 6 h.

### 2.5 Outcomes and operational definitions

The prespecified primary outcome was movement pain at 24 h after surgery, measured on the 0–10 numerical rating scale. Secondary analgesic outcomes comprised resting and movement pain at 12 and 48 h, the time-weighted mean movement-pain score from 12 to 48 h, movement pain of at least 4 at any scheduled assessment, cumulative sufentanil delivery at 24 and 48 h, effective patient-controlled demands, and rescue analgesia. The time-weighted pain score was calculated using the trapezoidal area under the pain-time curve divided by the observed interval. Rescue analgesia was defined as administration of an additional systemic analgesic for inadequately controlled postoperative pain beyond the scheduled multimodal regimen.

Recovery outcomes were time from completion of surgery to first mobilisation out of bed, first passage of flatus, first oral intake, urinary catheter removal, and first documented effective breastfeeding, together with postoperative length of stay. Safety outcomes included nausea or vomiting, pruritus, bradycardia, hypotension, excessive sedation, respiratory depression, early PCIA discontinuation, and postpartum haemorrhage of at least 1000 mL. Bradycardia was defined as a documented heart rate below 50 beats/min, and hypotension as a systolic blood pressure below 90 mmHg or hypotension requiring active treatment. Excessive sedation was defined as documented difficult arousal or sedation requiring additional clinical observation or intervention, whereas respiratory depression was defined as a respiratory rate below 8 breaths/min, clinically significant oxygen desaturation, or respiratory support attributed to opioid or sedative exposure. Nausea or vomiting and pruritus were counted when clinically documented and treated, and early PCIA discontinuation was defined as cessation before the planned 48-h period because of suspected treatment intolerance or an adverse event. Exploratory outcomes were postoperative platelet count, prothrombin time, activated partial thromboplastin time, fibrinogen, and D-dimer, each analysed with its corresponding preoperative value. Five-minute Apgar score and neonatal birthweight were prespecified as negative-control outcomes.

### 2.6 Statistical analysis, missing data, and additional analyses

Continuous variables were summarised as mean (standard deviation) or median (interquartile range), and categorical variables as number (percentage). The propensity score for receiving dexmedetomidine-containing PCIA was estimated using logistic regression including the 16 baseline covariates specified above, and overlap weights were derived from the estimated treatment probabilities; covariate balance was assessed using absolute standardised differences, with values below 0.10 considered acceptable. The primary outcome, movement pain at 24 h, was analysed using overlap-weighted linear regression with robust standard errors and reported as an adjusted mean difference with 95% confidence interval (13). Repeated pain measurements were examined using weighted linear mixed-effects models containing analgesia group, assessment time, and their interaction, with a participant-specific random intercept (14). Cumulative sufentanil delivery was analysed by weighted quantile regression, effective patient-controlled demands by negative binomial regression, and binary outcomes by modified Poisson regression with robust standard errors (15–17). Recovery times were modelled using log-normal accelerated failure-time models and reported as time ratios (18). Exploratory coagulation analyses, including baseline-adjusted D-dimer analyses, are described in the Supplement.

Outcome values were not imputed; outcome-specific analyses used available observations, and all available repeated pain measurements were retained in the mixed-effects models. Variable-specific baseline covariate missingness and outcome-specific analytic denominators are reported in Supplementary Table S1. Missing baseline covariates were handled using 20 multiply imputed datasets; the propensity score and overlap weights were re-estimated separately within each imputed dataset, analysis estimates were combined using Rubin's rules, and a complete-case analysis was performed as a sensitivity analysis (19, 20). Sensitivity analyses included 1:1 propensity-score matching, conventional multivariable adjustment, complete-case analysis, restriction to women receiving the complete standard PCIA formulation and pump programme, exclusion of intraoperative dexmedetomidine exposure, and an expanded propensity-score model additionally incorporating attending anaesthesiologist and calendar month (21). Exploratory subgroup analyses considered intrathecal morphine use, transversus abdominis plane block, and previous caesarean delivery; regimen-by-subgroup interaction terms were used to assess statistical evidence of heterogeneity in the association. Five-minute Apgar score and birthweight served as negative-control outcomes (22, 23). Exploratory coagulation sensitivity analyses are reported in the Supplement. Secondary and exploratory analyses were not adjusted for multiplicity and were interpreted as supportive or exploratory rather than confirmatory. All tests were two-sided, with *P* < 0.05 considered statistically significant. Analyses were conducted using R version 4.4.1.

## 3 Results

### 3.1 Cohort assembly and baseline covariate balance

Of 346 women screened, 42 were excluded and 304 met the eligibility criteria for the primary analysis. The cohort comprised 132 women who received dexmedetomidine-containing patient-controlled intravenous analgesia and 172 who received conventional analgesia (Figure 1). Movement pain at 24 h, the prespecified primary outcome, was available for all 304 women; pain assessments at 12 and 48 h were available for 299 (98.4%) and 296 (97.4%), respectively. Complete 48-h analgesia-pump records were available for 290 women. Baseline propensity-score covariates were complete for 292 women (96.1%); variable-specific covariate missingness and outcome-specific analytic denominators are reported in Supplementary Table S1. The propensity score-matched sensitivity cohort included 112 matched pairs.

Before weighting, the two analgesia groups showed modest differences in several clinical and perioperative characteristics. Women receiving dexmedetomidine-containing analgesia had a lower mean body mass index, shorter mean operative duration, and were more frequently nulliparous and exposed to intrathecal morphine. The largest absolute standardised difference was observed for operative duration (0.215), followed by body mass index (0.178), nulliparity (0.153), and previous caesarean delivery (0.141) (Table 1). After overlap weighting, all measured covariates had absolute standardised differences below 0.03, with a maximum value of 0.023. The first to 99th percentile of the overlap weights ranged from 0.16 to 0.79, and the weighted effective sample size was 287.6.

**Table 1:** Baseline, obstetric, anaesthetic, and perioperative characteristics before and after propensity-score overlap weighting.

Characteristic	Dexmedetomidine-containing analgesia (*n* = 132)	Conventional analgesia (*n* = 172)	Absolute standardised difference before weighting	Absolute standardised difference after weighting
Age, years	31.3 (4.3)	31.9 (4.6)	0.135	0.008
Body mass index, kg/m^2^	27.4 (3.2)	28.0 (3.5)	0.178	0.011
Gestational age, weeks	39.0 (0.7)	39.1 (0.8)	0.133	0.004
Nulliparous	76 (57.6%)	86 (50.0%)	0.153	0.006
Previous caesarean delivery	47 (35.6%)	73 (42.4%)	0.141	0.009
Gestational diabetes mellitus	27 (20.5%)	41 (23.8%)	0.081	0.005
Hypertensive disorder of pregnancy	12 (9.1%)	17 (9.9%)	0.028	0.003
American Society of Anesthesiologists physical status II	121 (91.7%)	155 (90.1%)	0.056	0.004
Combined spinal–epidural anaesthesia (vs. epidural anaesthesia)	71 (53.8%)	102 (59.3%)	0.111	0.012
Intrathecal morphine	36 (27.3%)	38 (22.1%)	0.121	0.015
Transversus abdominis plane block	29 (22.0%)	45 (26.2%)	0.098	0.007
Pre-existing chronic pain or regular analgesic use >3 months	5 (3.8%)	7 (4.1%)	0.014	0.002
Duration of surgery, min	46.5 (11.8)	49.2 (13.1)	0.215	0.023
Estimated blood loss, mL	350 [300–450]	380 [300–480]	0.122	0.016
Preoperative haemoglobin, g/L	118.2 (10.0)	116.9 (10.7)	0.126	0.010
Preoperative platelet count, ×10⁹/L	211 (51)	217 (54)	0.114	0.013

Data are mean (SD), median [IQR], or n (%). ASD denotes absolute standardised difference. Summary statistics are based on observed data; baseline covariate missingness is reported in Supplementary Table S1. All variables shown were included in the primary propensity-score model. Intrathecal morphine refers only to morphine administered intrathecally during combined spinal–epidural anaesthesia; epidural morphine was not used. An ASD <0.10 was considered acceptable covariate balance. The maximum ASD decreased from 0.215 before weighting to 0.023 after weighting.

### 3.2 Dexmedetomidine-containing analgesia was associated with lower movement pain through 24 h, with little residual difference at 48 h

Movement pain was lower in the dexmedetomidine-containing analgesia group at both 12 and 24 h after surgery. At 12 h, mean movement pain scores were 3.6 (SD 1.3) versus 4.1 (SD 1.4), corresponding to an overlap-weighted mean difference of −0.42 points (95% CI −0.72 to −0.12; *P* = 0.006). At 24 h, the prespecified primary outcome, mean scores were 3.1 (SD 1.2) and 3.7 (SD 1.3), respectively, with an adjusted mean difference of −0.51 points (95% CI −0.80 to −0.22; *P* < 0.001). Resting pain also differed modestly at 12 h (2.3 vs 2.6; mean difference −0.24, 95% CI −0.48 to 0.00; *P* = 0.048) and 24 h (2.0 vs. 2.3; mean difference −0.23, 95% CI −0.45 to −0.01; *P* = 0.042) (Table 2; Figures 2A–C).

**Table 2:** PCIA protocol, concomitant analgesia, and postoperative pain outcomes.

Characteristic or outcome	Dexmedetomidine-containing analgesia (*n* = 132)	Conventional analgesia (*n* = 172)	Overlap-weighted effect estimate (95% CI)	*P* value
Prescribed analgesia regimen				
Dexmedetomidine dose, μg	100	0	—	—
Sufentanil dose, μg	100	100	—	—
Dezocine dose, mg	10	10	—	—
Ondansetron dose, mg	16	16	—	—
Total pump volume, mL	100	100	—	—
Background infusion, mL/h	1.5	1.5	—	—
Patient-controlled bolus, mL	2.0	2.0	—	—
Lockout interval, min	20	20	—	—
Scheduled non-steroidal anti-inflammatory drug	111 (84.1%)	141 (82.0%)	—	—
Postoperative paracetamol	48 (36.4%)	56 (32.6%)	—	—
Postoperative pain outcomes				
Resting NRS at 12 h, *n* = 299	2.3 (1.1)	2.6 (1.2)	Mean difference −0.24 (−0.48 to 0.00)	0.048
Movement NRS at 12 h, *n* = 299	3.6 (1.3)	4.1 (1.4)	Mean difference −0.42 (−0.72 to −0.12)	0.006
Resting NRS at 24 h, *n* = 304	2.0 (1.0)	2.3 (1.1)	Mean difference −0.23 (−0.45 to −0.01)	0.042
Movement NRS at 24 h, primary outcome, *n* = 304	3.1 (1.2)	3.7 (1.3)	Mean difference −0.51 (−0.80 to −0.22)	<0.001
Resting NRS at 48 h, *n* = 296	1.4 (0.8)	1.5 (0.9)	Mean difference −0.07 (−0.25 to 0.11)	0.446
Movement NRS at 48 h, *n* = 296	2.3 (1.0)	2.5 (1.1)	Mean difference −0.16 (−0.39 to 0.07)	0.172
Time-weighted mean movement NRS, 12–48 h, *n* = 292	3.0 (0.9)	3.4 (1.0)	Mean difference −0.38 (−0.59 to −0.17)	<0.001
Movement NRS ≥4 at any assessment	53 (40.2%)	93 (54.1%)	Risk ratio 0.77 (0.63 to 0.94)	0.011

Data are mean (SD) or n (%). NRS denotes numerical rating scale, ranging from 0 to 10, with higher values indicating greater pain. The listed drug doses and pump settings define the institutional standard PCIA protocol. A sensitivity analysis restricted to the 251 women who received the complete standard base formulation and pump programme is reported in Supplementary Table S3. Negative mean differences and risk ratios below 1 indicate lower outcome values or frequencies in the dexmedetomidine-containing PCIA group.

**Figure 2:**
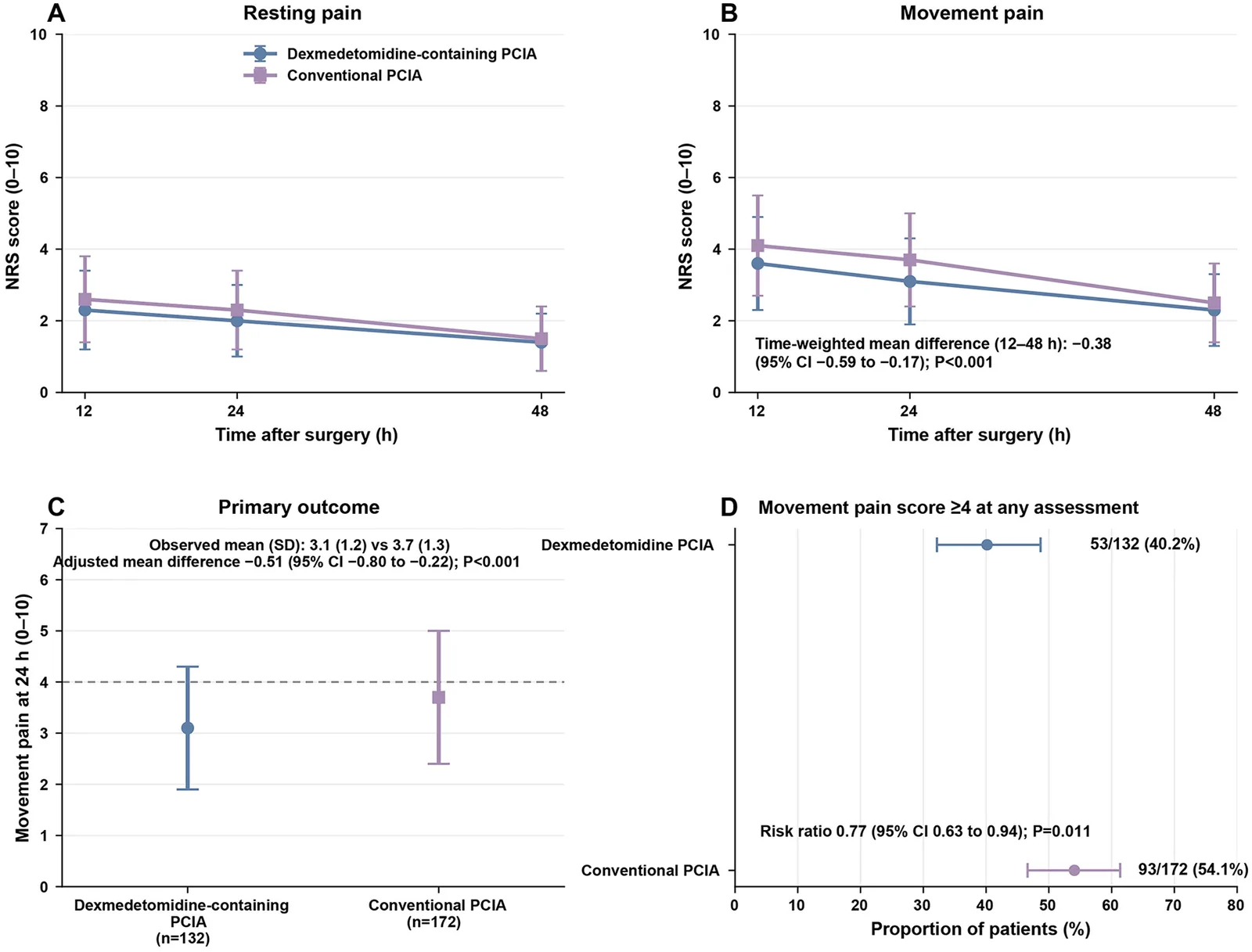
Postoperative pain during the first 48 h. **(A)** Resting numerical rating scale (NRS) scores at 12, 24, and 48 h after surgery. **(B)** Movement NRS scores at the same time points; the prespecified primary outcome was movement NRS at 24 h. In panels A and B, points show observed means and error bars show standard deviations. The time-weighted mean difference shown in panel B is the overlap-weighted estimate for 12–48 h. **(C)** Observed group means and standard deviations for movement pain at 24 h; the dashed horizontal line marks an NRS score of 4, and the adjusted mean difference is from the overlap-weighted primary model. **(D)** Proportion of women with movement NRS ≥4 at any postoperative assessment; points show observed proportions and horizontal lines show 95% confidence intervals. NRS ranges from 0 to 10, with higher scores indicating greater pain.

By 48 h, between-group differences were smaller and no longer statistically clear. Resting pain scores were 1.4 versus 1.5 (mean difference −0.07, 95% CI −0.25 to 0.11; *P* = 0.446), while movement pain scores were 2.3 versus 2.5 (mean difference −0.16, 95% CI −0.39 to 0.07; *P* = 0.172). Across the 12–48 h assessment period, the time-weighted mean movement pain score was 3.0 (SD 0.9) in the dexmedetomidine group and 3.4 (SD 1.0) in the conventional group, yielding a mean difference of −0.38 points (95% CI −0.59 to −0.17; *P* < 0.001). Movement pain of at least 4 at any assessment occurred in 53 of 132 women (40.2%) and 93 of 172 women (54.1%), respectively (risk ratio 0.77, 95% CI 0.63 to 0.94; *P* = 0.011) (Figure 2D).

### 3.3 Dexmedetomidine-containing analgesia was associated with lower analgesic requirements, while safety estimates remained imprecise

Analgesia-pump records were available for 296 women at 24 h and 290 at 48 h. Median cumulative sufentanil use was lower with dexmedetomidine-containing analgesia at both time points: 42 μg [IQR 39–47] versus 45 μg [41–51] at 24 h (adjusted median difference −3.0 μg, 95% CI −4.5 to −1.5; *P* < 0.001), and 84 μg [78–91] versus 90 μg [82–98] at 48 h (median difference −5.2 μg, 95% CI −7.8 to −2.6; *P* < 0.001). Among women with complete 48 h pump data, the median number of effective patient-controlled demands was 6 [3–9] and 9 [5–13], respectively (count ratio 0.76, 95% CI 0.65 to 0.89; *P* < 0.001). Rescue analgesia was required by 24 of 132 women (18.2%) in the dexmedetomidine group and 46 of 172 (26.7%) in the conventional group (risk ratio 0.72, 95% CI 0.52 to 0.99; *P* = 0.044) (Table 3; Figures 3A–C).

**Table 3:** Analgesic requirements, treatment-related events, and early postoperative recovery.

Outcome	Dexmedetomidine-containing analgesia (*n* = 132)	Conventional analgesia (*n* = 172)	Overlap-weighted effect estimate (95% CI)	*p* value
Analgesic requirements				
Cumulative sufentanil dose at 0–24 h, μg (*n* = 296)	42 [39–47]	45 [41–51]	Median difference −3.0 (−4.5 to −1.5)	<0.001
Cumulative sufentanil dose at 0–48 h, μg (*n* = 290)	84 [78–91]	90 [82–98]	Median difference −5.2 (−7.8 to −2.6)	<0.001
Effective patient-controlled demands (*n* = 290)	6 [3–9]	9 [5–13]	Count ratio 0.76 (0.65 to 0.89)	<0.001
Rescue analgesia	24 (18.2%)	46 (26.7%)	Risk ratio 0.72 (0.52 to 0.99)	0.044
Treatment-related events				
Nausea or vomiting	17 (12.9%)	31 (18.0%)	Risk ratio 0.76 (0.46 to 1.24)	0.273
Pruritus	7 (5.3%)	14 (8.1%)	Risk ratio 0.70 (0.30 to 1.62)	0.405
Bradycardia	7 (5.3%)	4 (2.3%)	Risk ratio 2.14 (0.69 to 6.65)	0.188
Hypotension	12 (9.1%)	12 (7.0%)	Risk ratio 1.29 (0.61 to 2.73)	0.505
Excessive sedation	4 (3.0%)	2 (1.2%)	Risk ratio 2.42 (0.51 to 11.5)	0.266
Respiratory depression	1 (0.8%)	1 (0.6%)	Risk ratio 1.27 (0.09 to 17.9)	0.858
Early PCIA discontinuation	4 (3.0%)	2 (1.2%)	Risk ratio 2.31 (0.50 to 10.6)	0.282
Postpartum haemorrhage ≥1000 mL	4 (3.0%)	7 (4.1%)	Risk ratio 0.77 (0.24 to 2.43)	0.656
Early postoperative recovery				
Time to first mobilisation, h (*n* = 301)	16.9 [14.2–20.5]	19.3 [16.0–23.2]	Time ratio 0.89 (0.84 to 0.95)	<0.001
Time to first flatus, h (*n* = 296)	25.8 [21.4–31.1]	28.9 [23.7–34.4]	Time ratio 0.92 (0.86 to 0.98)	0.012
Time to first oral intake, h (*n* = 298)	6.3 [5.5–7.6]	6.6 [5.6–8.0]	Time ratio 0.97 (0.92 to 1.03)	0.319
Time to urinary catheter removal, h (*n* = 300)	17.4 [15.6–19.5]	17.9 [16.0–20.1]	Time ratio 0.98 (0.94 to 1.03)	0.456
Time to first effective breastfeeding, h (*n* = 289)	3.3 [2.4–4.8]	3.7 [2.7–5.3]	Time ratio 0.92 (0.84 to 1.01)	0.082
Postoperative length of stay, days	4.0 [3.6–4.5]	4.1 [3.7–4.6]	Median difference −0.09 (−0.22 to 0.04)	0.174

Data are median [IQR] or n (%). Bradycardia was defined as documented heart rate <50 beats/min; hypotension as systolic blood pressure <90 mmHg or hypotension requiring active treatment; excessive sedation as clinically documented difficult arousal or sedation requiring additional observation or intervention; and respiratory depression as respiratory rate <8 breaths/min, clinically significant oxygen desaturation, or respiratory support attributed to opioid or sedative exposure. Nausea or vomiting and pruritus were counted when clinically documented and treated. Early PCIA discontinuation referred to cessation before the planned 48-h period because of suspected treatment intolerance or an adverse event. Events were identified from routine clinical records and were not independently adjudicated. Rare-event estimates are descriptive, and their wide confidence intervals preclude inference of safety equivalence. A time ratio below 1 indicates a shorter observed time to the recovery milestone.

**Figure 3:**
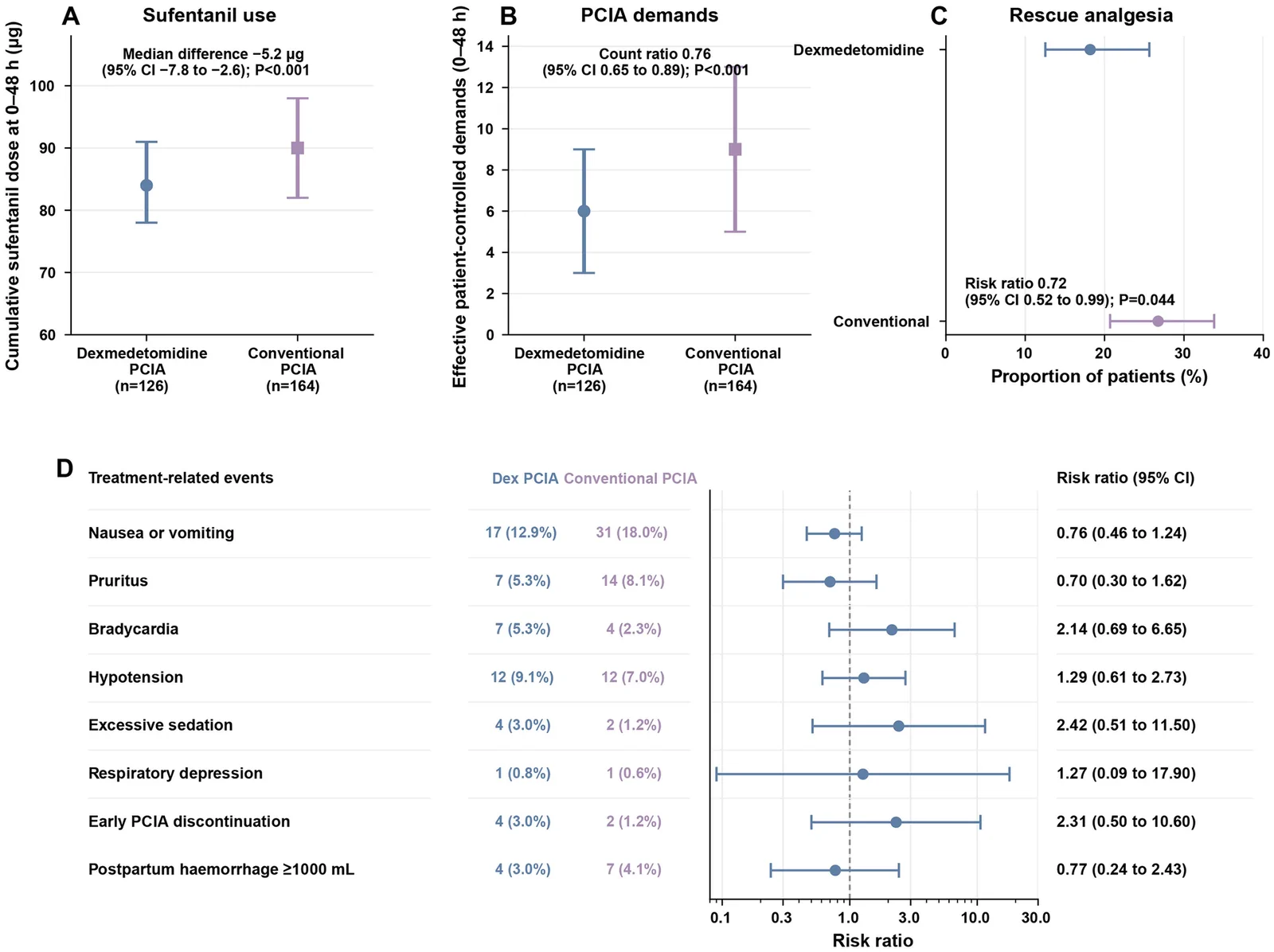
Analgesic requirements, rescue analgesia, and treatment-related events. **(A)** Cumulative sufentanil delivery during the first 48 postoperative hours among 290 women with complete pump records; points show medians and vertical intervals show interquartile ranges, and the adjusted median difference is from overlap-weighted quantile regression. **(B)** Effective patient-controlled demands during the first 48 h, shown as medians and interquartile ranges; the count ratio is from overlap-weighted negative-binomial regression. **(C)** Proportion of women receiving rescue analgesia; points show observed proportions and horizontal lines show 95% confidence intervals, and the risk ratio is from overlap-weighted modified Poisson regression. **(D)** Overlap-weighted risk ratios and 95% confidence intervals for treatment-related and perioperative events. The vertical reference line indicates a risk ratio of 1. Rare-event estimates are descriptive, and the wide confidence intervals do not establish safety equivalence.

Treatment-related events were uncommon, and most estimates were imprecise. Nausea or vomiting occurred in 17 of 132 women (12.9%) versus 31 of 172 (18.0%; risk ratio 0.76, 95% CI 0.46 to 1.24; *P* = 0.273). Bradycardia (5.3% vs. 2.3%), hypotension (9.1% vs. 7.0%), excessive sedation (3.0% vs. 1.2%), and early pump discontinuation (3.0% vs. 1.2%) were numerically more frequent with dexmedetomidine-containing PCIA, but the corresponding confidence intervals were wide and did not exclude clinically relevant increases in risk. Respiratory depression occurred in one woman in each group, and postpartum haemorrhage of at least 1000 mL occurred in 4 of 132 (3.0%) versus 7 of 172 (4.1%) (Figure 3D).

### 3.4 Selected early postoperative recovery times were shorter with dexmedetomidine-containing PCIA

Time to first mobilisation was available for 301 women and was shorter in the dexmedetomidine-containing analgesia group than in the conventional group: median 16.9 h [IQR 14.2–20.5] versus 19.3 h [16.0–23.2], corresponding to an overlap-weighted time ratio of 0.89 (95% CI 0.84 to 0.95; *P* < 0.001). First passage of flatus was documented in 296 women and occurred at 25.8 h [21.4–31.1] versus 28.9 h [23.7–34.4] (time ratio 0.92, 95% CI 0.86 to 0.98; *P* = 0.012). Differences in other recovery measures were smaller. Time to first oral intake was 6.3 h [5.5–7.6] versus 6.6 h [5.6–8.0] (time ratio 0.97, 95% CI 0.92 to 1.03; *P* = 0.319), and urinary catheter removal occurred at 17.4 h [15.6–19.5] versus 17.9 h [16.0–20.1] (time ratio 0.98, 95% CI 0.94 to 1.03; *P* = 0.456). First effective breastfeeding was numerically earlier with dexmedetomidine-containing analgesia, but the estimate remained compatible with no difference (3.3 h [2.4–4.8] vs. 3.7 h [2.7–5.3]; time ratio 0.92, 95% CI 0.84 to 1.01; *P* = 0.082). Postoperative length of stay was similar between groups (4.0 vs. 4.1 days; median difference −0.09 days, 95% CI −0.22 to 0.04; *P* = 0.174) (Table 3; Figures 4A,B).

**Figure 4:**
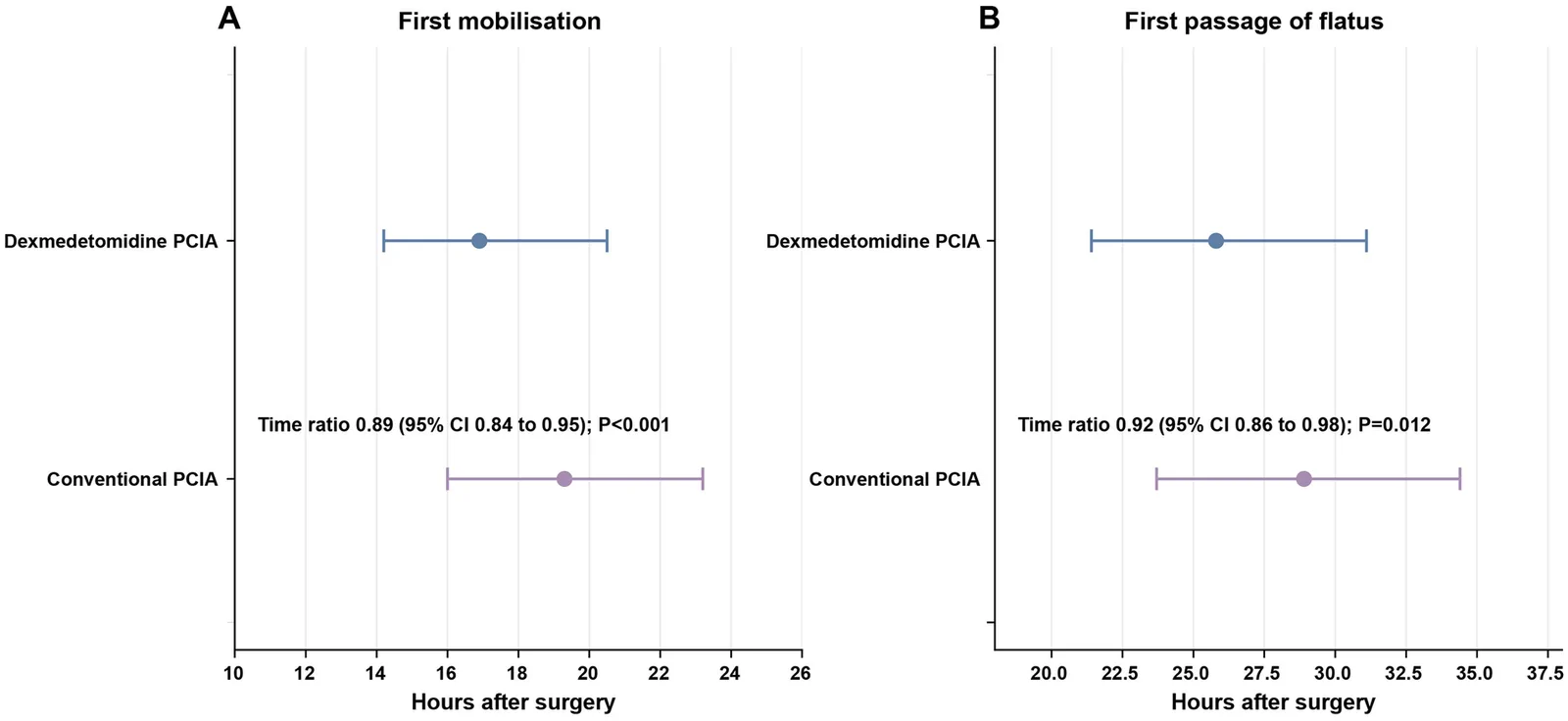
Early postoperative mobilisation and gastrointestinal recovery. **(A)** Time to first mobilisation among 301 women with available data. **(B)** Time to first documented passage of flatus among 296 women. Points show observed medians and horizontal intervals show interquartile ranges. Time ratios and 95% confidence intervals are from overlap-weighted log-normal accelerated failure-time models; a time ratio below 1 indicates a shorter observed time to the recovery milestone.

### 3.5 Exploratory coagulation analyses

Exploratory coagulation findings are presented in Supplementary Figure S2. The D-dimer association attenuated in sensitivity analyses and did not alter the interpretation of the primary clinical findings.

### 3.6 Sensitivity and subgroup analyses of the primary outcome

The association with lower movement pain at 24 h was consistent across alternative analytic specifications. The overlap-weighted estimate was −0.51 points (95% CI −0.80 to −0.22; *P* < 0.001), compared with −0.47 (95% CI −0.78 to −0.16; *P* = 0.003) after 1:1 propensity-score matching and −0.49 (95% CI −0.77 to −0.21; *P* < 0.001) after conventional multivariable adjustment. Results were similar in the complete-case cohort (−0.52, 95% CI −0.82 to −0.22; *P* < 0.001), among the 251 women receiving the complete standard PCIA formulation and pump programme (−0.44, 95% CI −0.75 to −0.13; *P* = 0.006), after excluding intraoperative dexmedetomidine exposure (−0.48, 95% CI −0.78 to −0.18; *P* = 0.002), and when attending anaesthesiologist and calendar month were added to the propensity-score model (−0.48, 95% CI −0.77 to −0.19; *P* = 0.001) (Supplementary Table S3; Figure 5A).

**Figure 5:**
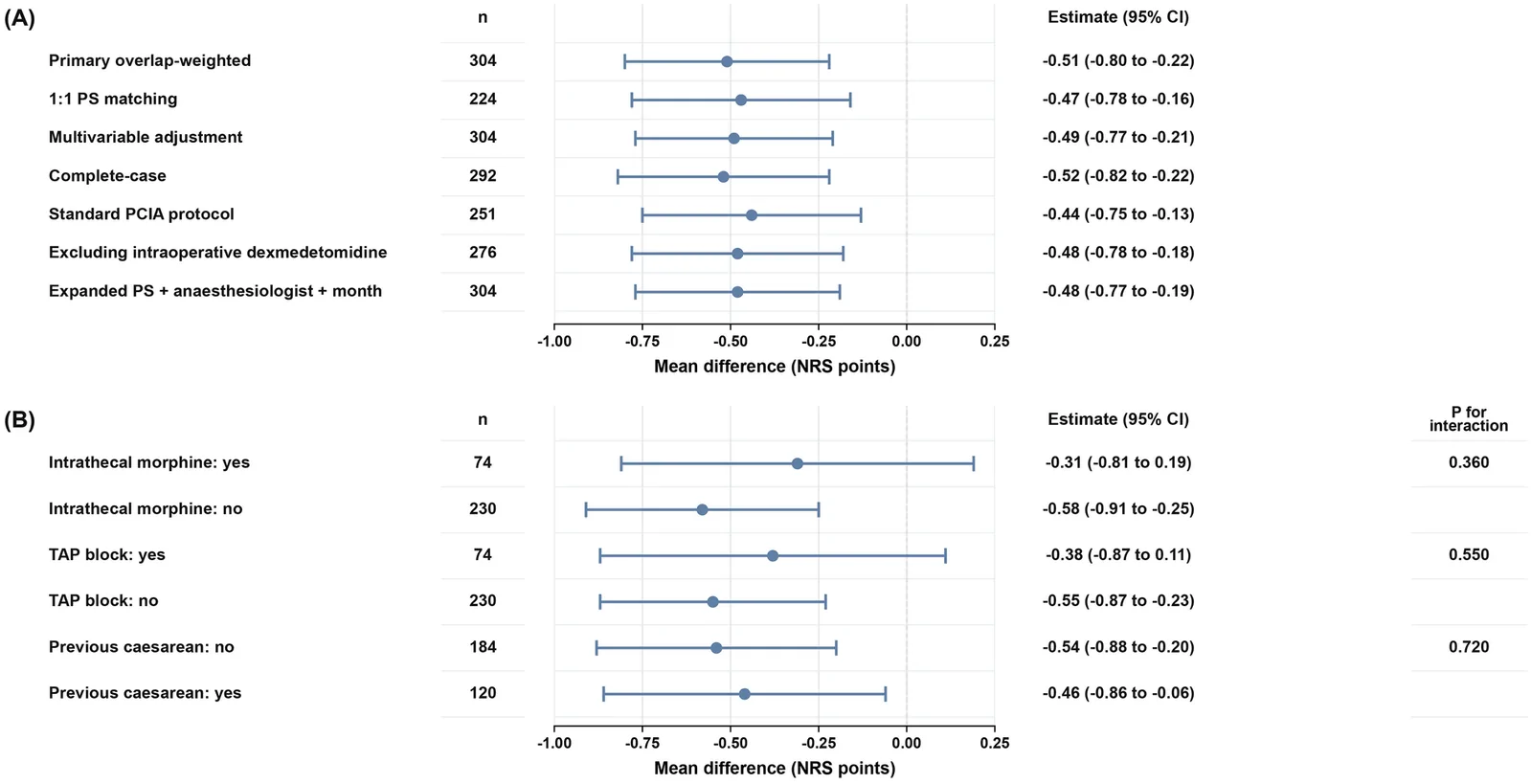
Sensitivity and subgroup analyses of movement pain at 24 h. **(A)** Primary overlap-weighted estimate and sensitivity analyses of the 24-h movement NRS outcome. The expanded propensity-score model additionally included attending anaesthesiologist and calendar month. **(B)** Exploratory subgroup analyses according to intrathecal morphine use, transversus abdominis plane block, and previous caesarean delivery; *P* values are from regimen-by-subgroup interaction terms. Points indicate mean differences and horizontal bars indicate 95% CIs. Mean differences are calculated as dexmedetomidine-containing minus conventional PCIA; negative values indicate lower movement pain in the dexmedetomidine-containing PCIA group. CI, confidence interval; NRS, numerical rating scale; PCIA, patient-controlled intravenous analgesia; PS, propensity score; TAP, transversus abdominis plane.

The estimate was numerically smaller among women who received intrathecal morphine (−0.31, 95% CI −0.81 to 0.19) than among those who did not (−0.58, 95% CI −0.91 to −0.25), but there was no statistical evidence of heterogeneity in the association (P for interaction=0.360). Similarly, the mean difference was −0.38 points (95% CI −0.87 to 0.11) among women receiving a transversus abdominis plane block and −0.55 points (95% CI −0.87 to −0.23) among those without a block (P for interaction=0.550). Among women without and with a previous caesarean delivery, the corresponding mean differences were −0.54 points (95% CI −0.88 to −0.20) and −0.46 points (95% CI −0.86 to −0.06), respectively (P for interaction=0.720). These subgroup analyses were exploratory. Five-minute Apgar score and birthweight did not differ materially between groups in the negative-control analyses (Supplementary Table S3; Figure 5B).

## 4 Discussion

In this retrospective cohort of 304 women undergoing elective term caesarean delivery, dexmedetomidine-containing patient-controlled intravenous analgesia was associated with modestly lower early movement pain and lower sufentanil delivery. The prespecified primary outcome, movement pain at 24 h, was lower by an adjusted mean of 0.51 points, and the direction of association was similar at 12 h and for the time-weighted movement-pain score from 12 to 48 h. Movement pain of at least 4 occurred in 40.2% of women receiving dexmedetomidine-containing analgesia and 54.1% receiving conventional analgesia. By 48 h, however, the between-group difference in movement pain had attenuated and was no longer statistically clear. This temporal pattern suggests that the observed association was concentrated in the early postoperative period rather than representing a sustained reduction in pain across all assessments.

The pain findings were accompanied by several internally consistent indicators of lower analgesic demand. Among women with complete pump records, median cumulative sufentanil delivery at 48 h was 84 μg versus 90 μg, with an adjusted median difference of −5.2 μg. Effective patient-controlled demands were also lower, with a count ratio of 0.76, and rescue analgesia was administered to 18.2% and 26.7% of women, respectively. The absolute opioid difference was not large, and the 0.51-point difference in movement pain should not be assumed to exceed a clinically important threshold because no minimum clinically important difference was prespecified. The concordant findings for movement-pain burden, actual pump delivery, patient-controlled demands, and rescue analgesia provide complementary support for the primary association, although they do not establish a causal treatment effect.

Previous studies have generally involved relatively small samples, standardised treatment protocols, and heterogeneous use of intrathecal morphine, dexmedetomidine doses, and non-opioid co-analgesia, limiting direct comparison with routine clinical practice (9, 24). The present study evaluated these associations in a routine clinical pathway in which intrathecal morphine, transversus abdominis plane block, and scheduled non-opioid medication were not uniformly applied. This pathway also differed from some contemporary enhanced-recovery protocols because standalone spinal anaesthesia was not used, neuraxial morphine and paracetamol were not universal, the PCIA programme included a continuous background infusion, and dezocine was included in the standard formulation. Accordingly, the estimated associations are most directly applicable to centres using similar postoperative analgesic pathways and may not be transportable without qualification to settings using routine neuraxial morphine and standardised scheduled non-opioid analgesia. The departmental standard PCIA formulations differed principally by the addition of dexmedetomidine 100 μg, but not all women received the complete standard base formulation and pump programme. The primary pain estimate remained similar across propensity-score matching, conventional multivariable adjustment, complete-case analysis, restriction to the standard PCIA protocol, exclusion of intraoperative dexmedetomidine exposure, and an expanded propensity-score analysis incorporating attending anaesthesiologist and calendar month. Although the point estimate was numerically smaller among women who received intrathecal morphine, the interaction analysis did not provide statistical evidence of heterogeneity in the association (P for interaction=0.360). The corresponding interaction for transversus abdominis plane block was also non-significant (P for interaction=0.550). These subgroup findings should therefore be regarded as hypothesis-generating rather than evidence that the association differs according to neuraxial morphine or regional-block use.

Earlier mobilisation and passage of flatus were observed with dexmedetomidine-containing analgesia. Median time to first mobilisation was 16.9 h versus 19.3 h, and time to first passage of flatus was 25.8 h versus 28.9 h. These secondary findings occurred in the same group in which lower pain and analgesic requirements were observed, but the observational data do not establish mediation or a direct recovery mechanism. Mobilisation and gastrointestinal recovery are also influenced by nursing schedules, catheter management, haemodynamic stability, patient preference, and ward routines (25). Consistent with this complexity, no clear differences were observed in first oral intake, urinary catheter removal, postoperative length of stay, or first effective breastfeeding.

Treatment-related events were uncommon, limiting precision. Nausea or vomiting was numerically less frequent with dexmedetomidine-containing analgesia, whereas bradycardia, excessive sedation, and early pump discontinuation were numerically more frequent. For example, bradycardia occurred in 5.3% versus 2.3%, and excessive sedation in 3.0% versus 1.2%, but the corresponding confidence intervals were wide. These data are insufficient to establish safety equivalence and do not exclude clinically important increases in uncommon adverse events. Exploratory coagulation findings are presented in the Supplement and were insufficiently robust to support clinical or mechanistic interpretation.

Several limitations are specific to the present data structure. Treatment selection was determined by the attending anaesthesiologist in routine clinical practice rather than by randomisation and could therefore have reflected provider preference or patient characteristics not fully captured by the measured covariates. Adjustment for attending anaesthesiologist and calendar month produced a similar primary estimate, and overlap weighting reduced the maximum absolute standardised difference from 0.215 to 0.023; nevertheless, residual and unmeasured confounding cannot be excluded. Pain assessments and recovery milestones were obtained from routine nursing records generated by multiple ward nurses who were not blinded to the clinical PCIA regimen, and the activity used to elicit movement pain may not have been identical across patients. Secondary outcomes had different denominators because pump logs, recovery times, and paired laboratory measurements were not uniformly available. The coagulation cohort was formed according to clinical testing availability, introducing potential selection bias. Safety events were too infrequent for precise risk estimation, and subgroup, negative-control, and laboratory analyses should be interpreted as exploratory. Finally, the single-centre setting, nine-month study period, restriction to elective term caesarean delivery, and institution-specific use of neuraxial morphine, regional blocks, basal PCIA infusion, dezocine, and non-opioid analgesia limit generalisability to emergency procedures, higher-risk obstetric populations, and hospitals using different multimodal analgesic pathways.

## 5 Conclusions

Among women undergoing elective term caesarean delivery, dexmedetomidine-containing PCIA was associated with modestly lower early movement pain and lower analgesic requirements, with shorter observed times to mobilisation and passage of flatus. Safety estimates for uncommon adverse events remained imprecise, and the data do not establish safety equivalence. These observational findings support further prospective evaluation within standardised multimodal analgesic pathways and should not be interpreted as establishing a causal treatment effect or supporting routine treatment selection.

## Data Availability

The original contributions presented in the study are included in the article/Supplementary Material, further inquiries can be directed to the corresponding authors.
