# Supplementary material for: Dexmedetomidine-containing patient-controlled intravenous analgesia after elective caesarean delivery: a retrospective cohort study
Source: Front Med (Lausanne). 2026 Oct 14;13:1991923. doi: 10.3389/fmed.2026.1991923 (PMC13713642; doi:10.3389/fmed.2026.1991923)
Supplement: Supplementary file 1 [file Table1.docx]

**Supplementary Table S1. Outcome definitions, assessment windows, and data completeness**

| **Variable** | **Operational definition or assessment window** | **Available data** |
| --- | --- | --- |
| **Movement pain at 24 h** | Pain during position change or mobilisation, recorded at 24±2 h | 304 (100.0%) |
| **Pain at 12 h** | Nearest valid resting and movement assessment within 12±2 h | 299 (98.4%) |
| **Pain at 48 h** | Nearest valid resting and movement assessment within 48±4 h | 296 (97.4%) |
| **Time-weighted mean movement pain** | Trapezoidal integration of available 12-, 24-, and 48-h assessments divided by observation time | 292 (96.1%) |
| **Actual pump delivery** | Recorded pump volume and successful patient-controlled demands through 48 h | 290 (95.4%) |
| **First mobilisation** | Time from completion of surgery to first standing or walking out of bed | 301 (99.0%) |
| **First flatus** | Time from completion of surgery to first documented passage of flatus | 296 (97.4%) |
| **First effective breastfeeding** | Time to first documented effective latch and feeding | 289 (95.1%) |
| **Any paired coagulation measurement** | Preoperative and postoperative values available for at least one prespecified marker | 261 (85.9%) |
| **Paired D-dimer measurement** | Preoperative and postoperative values reported using the same unit | 250 (82.2%) |

**Note:** Missing covariates were handled using multiple imputation. Outcomes were analysed using available observations.

**Supplementary Table S2. Propensity-score and outcome-model specifications**

| **Analysis component** | **Prespecified approach** | **Implementation or diagnostic result** |
| --- | --- | --- |
| Propensity-score estimation | Logistic regression including demographic, obstetric, anaesthetic, surgical, laboratory, and analgesia-related baseline variables | No complete separation; adequate overlap between treatment groups |
| Primary weighting | Overlap weights based on the probability of receiving the alternative treatment | Maximum absolute standardised difference decreased from 0.215 to 0.023 |
| Weight diagnostics | Weight distribution, effective sample size, and assessment of extreme observations | First to 99th percentile, 0.16–0.79; effective sample size, 287.6 |
| Repeated pain outcomes | Linear mixed-effects model including treatment, time, treatment-by-time interaction, and a participant-specific random intercept | Prespecified primary contrast was movement pain at 24 h |
| Sufentanil consumption | Quantile regression with robust variance estimation | Results reported as adjusted median differences |
| Patient-controlled demands | Negative-binomial regression with robust variance estimation | Results reported as count ratios |
| Binary outcomes | Modified Poisson regression with robust variance estimation | Results reported as risk ratios |
| Recovery times | Log-normal accelerated failure-time models | Results reported as time ratios |
| Coagulation outcomes | Postoperative value modelled with treatment and the corresponding preoperative value | D-dimer was logarithmically transformed |
| Missing covariates | Twenty multiply imputed datasets combined using Rubin’s rules | Complete-case analyses were used for sensitivity assessment |

**Note:** Outcome models incorporated overlap weights unless otherwise stated.

**Supplementary Table S3. Subgroup, sensitivity, and negative-control analyses**

| **Analysis** | **Sample size** | **Adjusted estimate (95% CI)** | **p value** | **Interaction p value** |
| --- | --- | --- | --- | --- |
| **Movement pain at 24 h: sensitivity analyses** |  |  |  |  |
| **Overlap-weighted primary analysis** | 304 | Mean difference −0.51 (−0.80 to −0.22) | <0.001 | — |
| **One-to-one propensity-score matching** | 224 | Mean difference −0.47 (−0.78 to −0.16) | 0.003 | — |
| **Conventional multivariable adjustment** | 304 | Mean difference −0.49 (−0.77 to −0.21) | <0.001 | — |
| **Complete-case analysis** | 292 | Mean difference −0.52 (−0.82 to −0.22) | <0.001 | — |
| **Standard base PCIA formulation and pump settings** | 251 | Mean difference −0.44 (−0.75 to −0.13) | 0.006 | — |
| **Excluding intraoperative dexmedetomidine** | 276 | Mean difference −0.48 (−0.78 to −0.18) | 0.002 | — |
| **Movement pain at 24 h: prespecified subgroups** |  |  |  |  |
| **Intrathecal morphine used** | 74 | Mean difference −0.31 (−0.81 to 0.19) | 0.225 | 0.360 |
| **Intrathecal morphine not used** | 230 | Mean difference −0.58 (−0.91 to −0.25) | <0.001 |  |
| **No previous caesarean delivery** | 184 | Mean difference −0.54 (−0.88 to −0.20) | 0.002 | 0.720 |
| **Previous caesarean delivery** | 120 | Mean difference −0.46 (−0.86 to −0.06) | 0.025 |  |
| **Postoperative D-dimer: sensitivity analyses** |  |  |  |  |
| **Overlap-weighted paired analysis** | 250 | Geometric mean ratio 0.91 (0.83 to 0.99) | 0.039 | — |
| **Propensity-score matched paired analysis** | 190 | Geometric mean ratio 0.93 (0.84 to 1.03) | 0.174 | — |
| **Sampling restricted to 24±6 h** | 222 | Geometric mean ratio 0.92 (0.83 to 1.01) | 0.086 | — |
| **Negative-control outcomes** |  |  |  |  |
| **Five-minute Apgar score** | 304 | Mean difference 0.00 (−0.04 to 0.04) | 0.941 | — |
| **Birthweight, g** | 304 | Mean difference 10 (−47 to 67) | 0.731 | — |

**Note:** Negative pain differences favour dexmedetomidine-containing analgesia. The D-dimer association was less robust than the primary pain finding.

**Supplementary Figure S1. Propensity-score diagnostics and covariate balance.**

**(A)** Kernel-density distributions of overlap weights in the two analgesia groups.
**(B)** Absolute standardised differences before and after propensity-score overlap weighting.
**(C)** Propensity-score distributions before matching and after 1:1 propensity-score matching.
**(D)** Absolute standardised differences before and after matching. Dashed vertical lines in panels B and D indicate the prespecified balance threshold of 0.10.

Supplementary Figure S2

**Supplementary Figure S2. Additional perioperative coagulation profiles and postoperative sampling-time diagnostics.**

**(A)** Paired preoperative and postoperative platelet counts.
**(B)** Paired preoperative and postoperative prothrombin times.
**(C)** Paired preoperative and postoperative activated partial thromboplastin times. Violin plots show the distributions, box plots show medians and interquartile ranges, and thin lines connect paired measurements.
**(D)** Kernel-density distributions of postoperative D-dimer sampling times according to analgesia group. The shaded region denotes the prespecified 24 ± 6 h sampling window.
